# Siamese network with change awareness for surface defect segmentation in complex backgrounds

**DOI:** 10.1038/s41598-025-94733-4

**Published:** 2025-04-07

**Authors:** Biyuan Liu, Sijie Luo, Huiyao Zhan, Yicheng Zhou, Zhou Huang, Huaixin Chen

**Affiliations:** 1 School of Resources and Environment, University of Electronic Science and Technology of China, Chengdu, 611731 China; 2 South China Normal University, Shanwei, 516600 China; 3 Sichuan Changhong Electric Co., Ltd., Mianyang, 621000 China

**Keywords:** Surface defect segmentation, Change-aware decoder, Siamese network, Contrastive learning, Transformer-based encoder, Characterization and analytical techniques, Design, synthesis and processing, Electronic devices

## Abstract

Despite the significant advancements made by deep visual networks in detecting surface defects at a regional level, the challenge of achieving high-quality pixel-wise defect detection persists due to the varied appearances of defects and the limited availability of data. To address the over-reliance on defect appearance and enhance the accuracy of defect segmentation, we proposed a Transformer-based Siamese network with change awareness, which formulates the defect segmentation under a complex background as change detection to mimic the human inspection process. Specifically, we introduced a novel multi-class balanced contrastive loss to guide the Transformer-based encoder, enabling it to encode diverse categories of defects as a unified, class-agnostic difference between defective and defect-free images. This difference is represented through a distance map, which is then skip-connected to the change-aware decoder, assisting in localizing pixel-wise defects. Additionally, we developed a synthetic dataset featuring multi-class liquid crystal display (LCD) defects set within a complex and disjointed background context. In evaluations using our proposed and two public datasets, our model outperforms leading semantic segmentation methods while maintaining a relatively compact model size. Furthermore, our model achieves a new state-of-the-art performance compared to semi-supervised approaches across various supervision settings. Our code and dataset are available at https://github.com/HATFormer/CADNet.

## Introduction

Surface defect inspection is crucial in manufacturing to prevent potential quality issues, economic loss, and even safety hazards. These defects can manifest in various forms, such as dirt, spots, and fractures. They are commonly found across various industrial products, encompassing steel^1,2^, LED^3^, and magnetic tile^4^. Unlike semantic objects, the surface defects generally do not have a regular shape, clear interpretation, or continuous context with the background, which complicates the application of empirically designed methods^5^. To facilitate the automation of defect inspection, deep learning-based approaches have been widely applied in multi-level defect detection. (1) Image-level classification in earlier works focus on classifying whether an image contains defects or not, without giving a specific pixel-wise location^6–8^. In SegNet^1^ and its variants^9,10^, pixel-level annotations are introduced as auxiliary information to the network yet ultimately output the binary classification results. (2) Defect localization at fuzzy level refers to obtaining a relatively fine-grained output without pixel-wise supervision. For instance, the class activation map^11^ refers to a technique that enables the visualization of the regions within an image that a convolutional neural network (CNN) focuses on when making a classification decision. It is utilized for locating the blurry LED defects^3^ and industrial anomalies^12^ with image-level supervision. The methods based on non-defective sample modeling^13–15^, focus on modeling the distribution of defect-free data in the training phase, and subsequently assess the deviations in the distribution between anomaly and normal samples. Reconstruction-based anomaly detection approaches^16–18^ aim to reconstruct normal data instances based on similarity metrics and then locate and identify anomalies through pixel-level differences between the anomalous and reconstructed data^19^.

While these methods do not necessitate a substantial volume of training data, the absence of meticulous supervision results in imprecise pixel-level predictions. (3) Fine-grained segmentation has been increasingly applied for defect detection^2,20–23^. There exists a paradox between striving for zero defect manufacturing^24^ and the availability of sufficient defective samples. To alleviate the shortage of pixel-label annotations, various studies have introduced additional priors, including visual saliency^25^, repeat pattern analysis^26^, and interactive click^22^. Additionally, these studies have embraced semi-supervised techniques such as pseudo labeling^5,27^ and consistency regularization^28^, to enhance their approaches further.

**Fig. 1:**
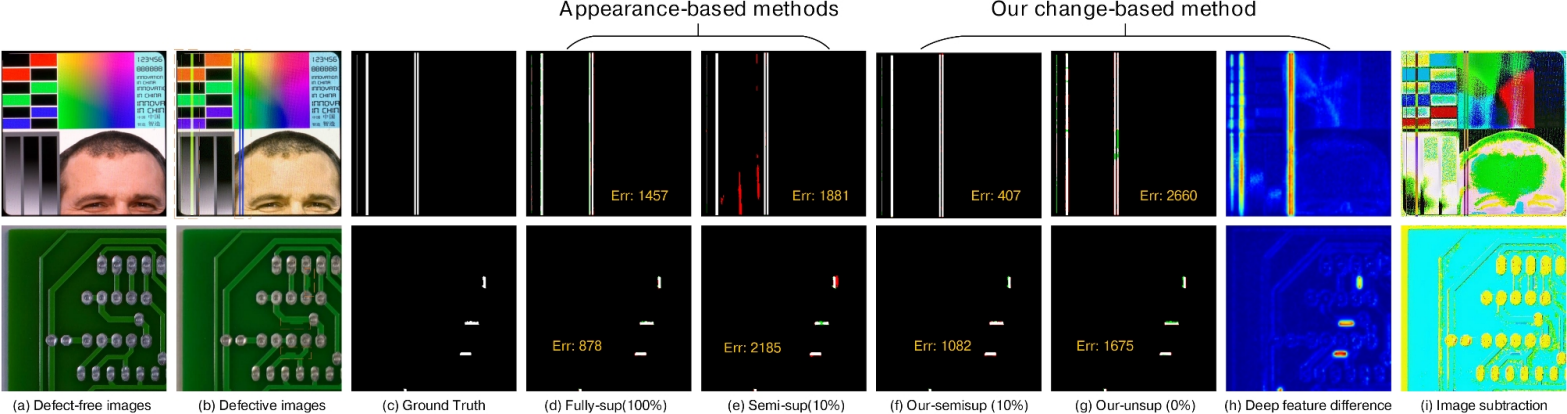
The examples illustrate how our change-based and appearance-based methods have segmented defects in fully-supervised, semi-supervised, and unsupervised settings. The results in column (**d**) are derived from SegFormer^29^. The outcomes in column (**e**) originate from UAPS^5^. In the prediction maps, green signifies missed detections, and red indicates erroneous detections. The term “Err” quantifies the total of these errors. Our model outperforms semi-supervised methods and achieves competitive outcomes using only 10% of the training samples compared to the fully-supervised model.

A review of existing literature reveals that most defect detection methods remain focused on image-level classification^6–8^ and fuzzy-level localization^3,11,12^, primarily due to the difficulty of obtaining pixel-wise labels in real-world production environments. Some studies have attempted to develop fine-grained segmentation methods using semi-supervised techniques^5,22,26,27^. However, defect segmentation in industrial products such as LCD screens, PCBs, and printed materials remains challenging due to complex backgrounds, irregular defect shapes, and a lack of sufficient labeled samples. The appearance prior refers to pre-existing knowledge or assumptions about the visual characteristics of defects^30^. This typically involves relying on specific patterns, shapes, textures, or other visual cues learned from training data to locate and classify defects. However, these aforementioned defect segmentation methods that locate defects based on appearance priors are not reliable due to the inherent contradiction between data scarcity and the diverse appearance of defects (see Fig. 1 (d)(e)). Limited defect samples can yield a skewed representation of the actual data distribution, subsequently leading to deteriorated generalization performance in these appearance-based methods^5^. It should be emphasized that locating defects based on their visual characteristics in industrial products, such as printed circuit boards (PCBs), liquid crystal displays (LCDs), and printed publications, presents a substantial challenge. The complex and occasionally ambiguous patterns of the background can obscure these defects, consequently increasing the complexity of their detection (see Fig. 1 (a)(b)(c)).

Our motivation to transform defect detection into a change detection problem is based on two self-evident facts: (1) Obtaining defect-free samples is considerably easier than acquiring defect images. (2) Defect regions essentially correspond to the differences between defect-free and defective samples. Identifying defective regions proves challenging without a clean reference, even for human observers. In this regard, we propose an accurate defect segmentation method based on data simulation and change feature modeling. This approach is particularly effective for surface defects with relatively steady but complex background patterns, such as PCB, LCD, and printed publications.

Several research gaps persist in current studies. First, there is a lack of sufficient labeled samples for training fully-supervised models that can achieve high-accuracy defect segmentation. Second, achieving high-accuracy segmentation with limited labeled data remains challenging due to the appearance-based nature of most segmentation methods. Third, the fundamental concept that defect detection is primarily based on sample comparison has not been fully acknowledged. As mentioned earlier, there is a need to design a method that leverages easily obtainable defect-free samples and performs defect segmentation based on a change detection mechanism. Additionally, label efficiency and computational efficiency are key considerations when designing the model. Addressing these issues is crucial to meet the requirements of real-world production environments and to bridge the gaps in current research. To this end, we propose a defect segmentation method that enables deep feature differencing between defective and defect-free industrial images. This method is characterized by the parallel modeling of defective and defect-free images, contrastive feature learning, and change-aware decoding. Specifically, we propose a novel change-aware Siamese network with a change attention mechanism to solve pixel-wise defect detection. In the encoding stage, a Transformer-based Siamese network constrained by multi-class balanced contrastive loss (BCL) is employed to extract the difference features between the defect-free and the defective samples. Then, the hierarchical Siamese feature pairs are fused by multi-stage subtraction and upsampled to a high resolution. In the decoding stage, the feature distance map is skip-connected to the decoder and acts as a change region attention to assist in locating the pixel-wise defects. In contrast to directly modeling the defect appearance, our proposed method models the defects as differences between defect-free and defective images. Interestingly, we find that this structure exhibits considerable generalization with limited labeled samples, as shown in Fig. 1 (f)(g). This can be attributed to our method of learning the deep feature difference shown in Fig. 1 (h). For comparison, Fig. 1 (i) shows the noisy results of directly performing image subtraction.

Furthermore, the community dedicated to surface defect detection requires a challenging dataset. The predominance of smaller datasets obstructs the thorough evaluation of current models. For instance, the average precision for commonly utilized datasets such as KolektorSSD^10^, DAGM2007^9^, and Severstal-Steel^10^ has attained the levels of 100$\%$, 100$\%$, and 98.7$\%$, respectively. Given the rapid ascension of LCDs as a leading display technology with extensive use in computers and mobile phones, we introduce a novel dataset aimed at enhancing LCD defect detection. To summarize, our contributions are as follows:

- We propose a change-aware Siamese network for defect segmentation. The modeling mechanism relies on changing features between clean and defective images instead of defect appearance, enabling synthetic data supervision and unseen class generalization.
- To simulate stable but complex background surface defects and to further benefit the field of LCD defect detection, we introduce a synthetic LCD defect dataset named SynLCD, which is utilized as a benchmark for comparison with other segmentation methods.
- The experiments conducted on the SynLCD, PKU-Market-PCB^31^, and MvTec-AD^16^ datasets show that our network outperforms the current mainstream appearance-based segmentation methods. Additionally, a comparison with five state-of-the-art (SOTA) semi-supervised segmentation methods underscores our model’s superiority across various supervision levels.

## Related works

In this section, we introduce surface defect detection at various levels of detection granularity, along with change detection methods. The work most relevant to our study involves anomaly detection methods based on reconstruction and differencing. These methods identify the approximate location of general surface defects using a differencing process between reconstructed and input images. In contrast, we employ deep feature change detection instead of simple differencing in the image space. Our focus is on precise segmentation in scenarios where defects can be subtle and potentially obscured during the reconstruction process. This focus is crucial to maintaining our primary emphasis on the core issue.

### Surface defect detection

**Image-label detection.** Masci *et al.*^6^ applied CNN to steel surface defect detection, highlighting CNN’s superiority over manual features. Faghih-Roohi *et al.*^7^ explored the impact of network complexity on defect detection performance. Racki *et al.*^8^ introduced a compact CNN for detecting synthetic textured anomalies by incorporating auxiliary segmentation labels alongside the classification task. SegNet^1^ refined this approach by merging the distinct stages of segmentation and classification into an end-to-end training framework. Božič *et al.*^10^ embarked on an exploration of the impact of varying levels of supervision, from weak to full, on the accuracy of defect classification. Moreover, the general principles of feature extraction and handling complex data distributions in^32^ may offer valuable insights in collecting labeled training data efficiently and at a lower cost for defect classification. The work on^33^ emphasized the power of combining multiple models to improve accuracy, including preprocessing, feature extraction with multiple feature descriptors, and classification using various classifiers. Despite these advancements, early deep learning-based research primarily focused on image-level defect detection, with limited attention to pixel-wise defect localization.

**Fuzzy and region-level detection.** Limited by the pixel-wise annotations in the anomaly detection task, some studies seek to consult the weak-supervised^3,12^ and unsupervised learning^19^. A class activation map^11^ is widely used to indicate the potential anomalous regions within an image with only image-level hints^3,12^. However, this merely eases annotation labor but fails to address the fundamental issue of data scarcity. On the other hand, the wealth of defect-free data significantly prompts the advancement of non-defective modeling and reconstruction-based methods. The *non-defective modeling* focuses on building an embedding model of normal samples and identifying the anomaly instances by measuring their deviation from the latent space. Defects are fuzzily spotted by patch-wise representation (*e.g.*, PatchCore^13^ and ReconPatch^14^), receptive field upsampling^34^, and gradient back-propagation in a normalizing-flow-based model^15^. The *reconstruction-based model* is typically trained to reconstruct defect-free samples and identify anomalies, while it fails to generate instances. The autoencoder^16^ and generative-adversarial network (GAN)^17^ are commonly employed in the reconstruction process. A straightforward differencing process between the input and reconstructed samples is applied to obtain defect regions, such as the element-wise square distance in EfficientAD^18^. However, a common issue is the occurrence of false-positive detections triggered by imprecise reconstructions of normal images. To sum up, due to the absence of pixel-wise annotations for these methods, it remains unclear which image points are anomalies, leading to indistinct detection results.

**Pixel-wise detection.** Recently, there has been a growing focus on pixel-level defect detection extended **from** semantic segmentation models. He *et al.*^21^ proposed locating wood defects by adopting the FCN architecture^35^. Huang and Xiang^26^ adapted the DeepLab v3+ architecture^36^ with minor modifications for the fabric defect segmentation. Du *et al.*^37^ extended the U-Net^38^ into a two-stream structure for segmenting defects in X-ray images. More recently, attention mechanisms have been employed for modeling local and global contextual dependencies. Dong *et al.*^23^ proposed segmenting steel surface defects with global context attention. Yeung *et al.*^39^ refined SegFormer^40^ with a boundary-aware module for Transformer-based defect segmentation. The work on^41^ integrated self-attention with dual encoder-decoder for biomedical segmentation with a noisy background. The importance of multi-scale feature learning was emphasized in^42^ for improving segmentation accuracy. Defect segmentation enhances understanding of defective samples but is constrained by the cost of fine-grained labels.

Therefore, some recent studies resort to semi-supervised techniques such as pseudo labeling^5,27^ and consistency regularization^28^. Pseudo-labeling methods^43,44^ generate pseudo-labels for unlabeled samples via a pre-trained network, potentially enhancing model performance with these additional training signals. However, the predictive noise in unlabeled samples can compromise pseudo-label quality, thereby constraining their utility. Consistency regularization posits that model predictions for unlabeled samples should remain consistent under controlled perturbations, aiming to minimize prediction discrepancies in different scenarios. Various heuristics have been introduced for consistency regularization, such as co-training^45^, mean teacher^46^, and multi-head prediction uncertainty^5^. Additionally, the active learning techniques explored in^47^ also share a similar philosophy of minimizing annotation costs while improving model performance. We provide a comparison between these semi-supervised methods and our change-modeling architecture given limited labeled samples in Table 6.

### Change detection

Image change detection is designed to identify pre-defined differences between the images captured at different times^48^. The primary challenge in change detection lies in differentiating semantic changes from noisy alterations, including variations in illumination, saturation changes, and disturbances from irrelevant backgrounds.^49^. It is widely applied in handwritten signature verification^50^, street scene^51^, and remote sensing change detection^48^. In ChangChip^52^, surface defects in PCB are identified through manual image registration and comparison. However, it entails prolonged preprocessing times and necessitates hyperparameter fine-tuning for image subtraction. Zagoruyko *et al.*^53^ pioneered the application of CNN for image comparison. Daudt *et al.*^54^ further developed an FCN-based Siamese architecture to enable arbitrary-sized image change detection. Several studies^51,55^ have concentrated on introducing contrastive loss^56^, a pivotal aspect for minimizing the distance of unchanged feature pairs while maximizing the distance of changed feature pairs. However, these contrastive approaches are primarily designed for binary changes and cause imbalance attention for different change categories, as illustrated in Fig. 7.

In our research context, the most relevant studies are background reconstruction methods^57,58^. These innovative works reconstruct flawless images from unlabeled data and employ a differential mapping technique between the original and reconstructed images to obtain the final segmentation map. However, the quality of the reconstructed image and image-level differencing becomes their bottlenecks.

## Method

### Problem definition: appearance-modeling vs. change-modeling

Industrial materials like LCD, PCB, and printed products (*e.g.*, books, drawings, and trademarks) exhibit relatively consistent appearances and surface patterns when they are defect-free. Based on this observation, we simplify the formation process of surface defect images, represented as $x_{ng}$ (where “ng” stands for “not good”). This involves overlaying a standard clean image $x_\text {ok}$, with $x_\text {defect}$ in a specific manner, followed by a global nonlinear transformation. This process can be formulated as:

1 $$\begin{aligned} x_\text {ng} = \sigma (x_\text {ok}\boxplus {x_\text {defect}}), \end{aligned}$$

where $\sigma$ represents a nonlinear global transformation (*e.g.*, material batch differences, aging, lighting, and imaging distortion), $\boxplus$ indicates a specific overlaying way (*e.g.*, corrosion, breakage, mixing, and direct covering). For the classical segmentation paradigms, the model $f'$ identifies defect objects based on their appearance and context, which can be formulated according to the assumption of equation (1) as:

2 $$\begin{aligned} {\hat{x}}_\text {defect} = f'(x_\text {ng}) =f'(\sigma (x_\text {ok}\boxplus {x_\text {defect}})). \end{aligned}$$

It implies that the model $f'$ is required to separate ${\hat{x}}_\text {defect}$ from complex background $x_\text {ok}$ under nonlinear interference $\sigma$. However, the background content may closely resemble defects, as depicted in Fig. 5 (g), rendering the distinction based on defect appearance unreliable. We aim to model the defect in defective images as different from defect-free ones, which is:

3 $$\begin{aligned} \begin{aligned} {\hat{x}}_\text {defect}&=f(x_\text {ng},\hat{x_\text {ok}}), \\&=\sigma ({{x}_\text {ok}}\boxplus {{x}_\text {defect}})\boxminus {\hat{{x}_\text {ok}}}. \end{aligned} \end{aligned}$$

In the change-modeling paradigm, the model learns a deep subtraction function $\boxminus$, overcoming limitations associated with defect appearance. The disturbance of the nonlinear transformation $\sigma$ and complex background is mitigated **by** the easily obtainable defect-free image ${\hat{x}}_\text {ok}$.

### Change-aware Siamese network

**Fig. 2:**
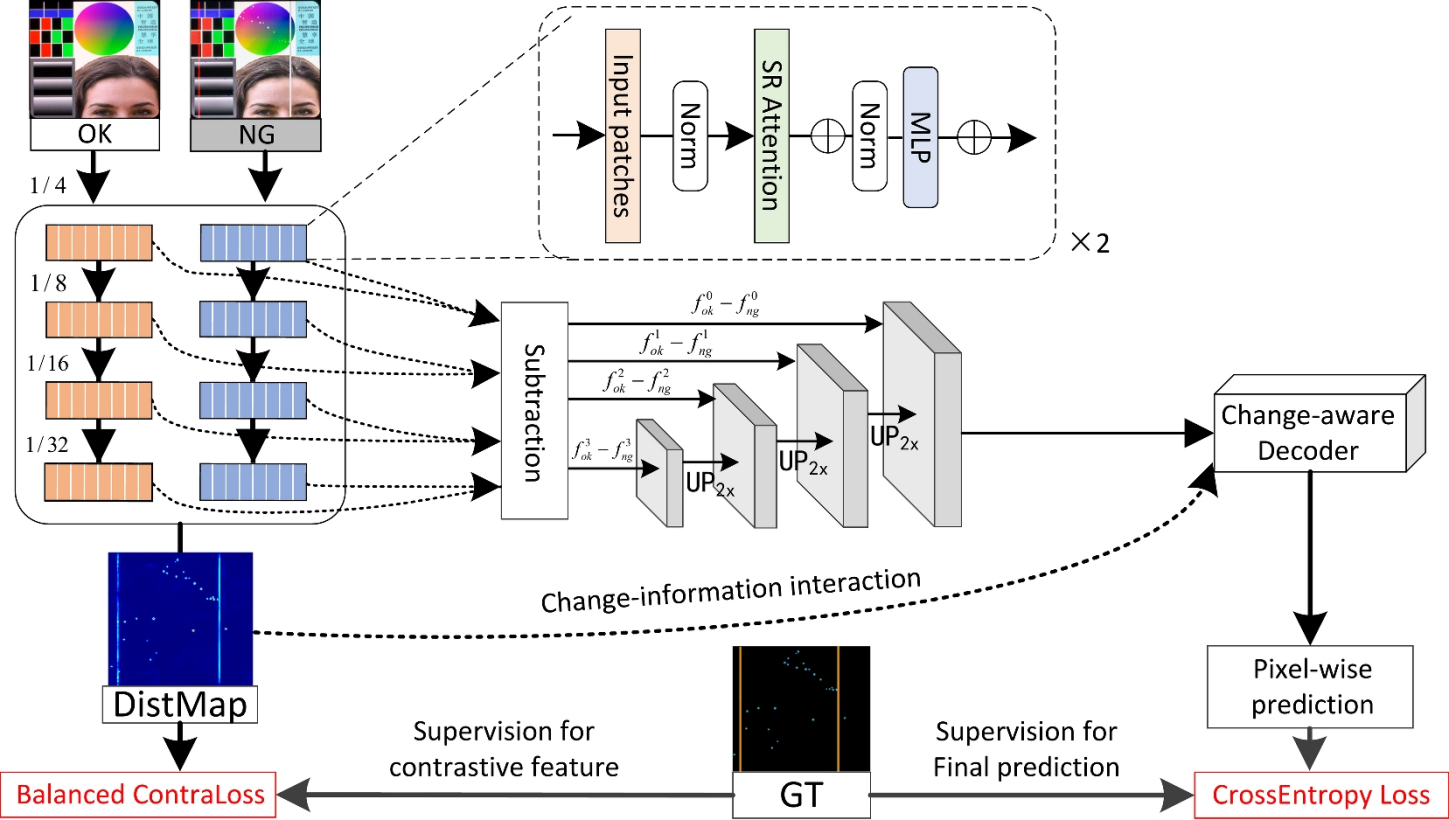
The pipeline of our Transformer-based change-aware defect detection network, CADNet, is designed to accept an image under inspection (NG) and a defect-free reference image (OK) for deep change modeling. The contrastive feature encoder, comprising a Siamese four-stage Transformer, generates a deep feature distance map (DistMap). The change-aware decoder leverages the DistMap to facilitate accurate defect localization. The network is trained using a cross-entropy loss and a multi-class balanced contrastive loss. Note that the ground truth used is a multi-class segmentation map.

Fig. 2 depicts our pipeline of change-aware Siamese network. The contrastive encoder extracts deep feature differences between the defective and defect-free samples. The change-aware decoder incorporates change information from the encoder to assist defect localization. The feature distance (DistMap) is used for change information interaction between the encoder and decoder. Specifically, the encoder contains an efficient Transformer-based backbone with four Transformer blocks^29,59^ using shared weights. Then, the hierarchical features are fused via multi-stage subtraction and upsampled to high resolution before decoding. In the decoding stage, the DistMap is used to introduce change information for locating pixel-wise defects. The whole network is supervised by two loss functions, where the cross-entropy loss evaluates the similarity between the predictions and the corresponding ground truth, while the balanced contrastive loss distinguishes the features of defective regions from those of defect-free regions.

#### Contrastive feature encoder

**Fig. 3:**
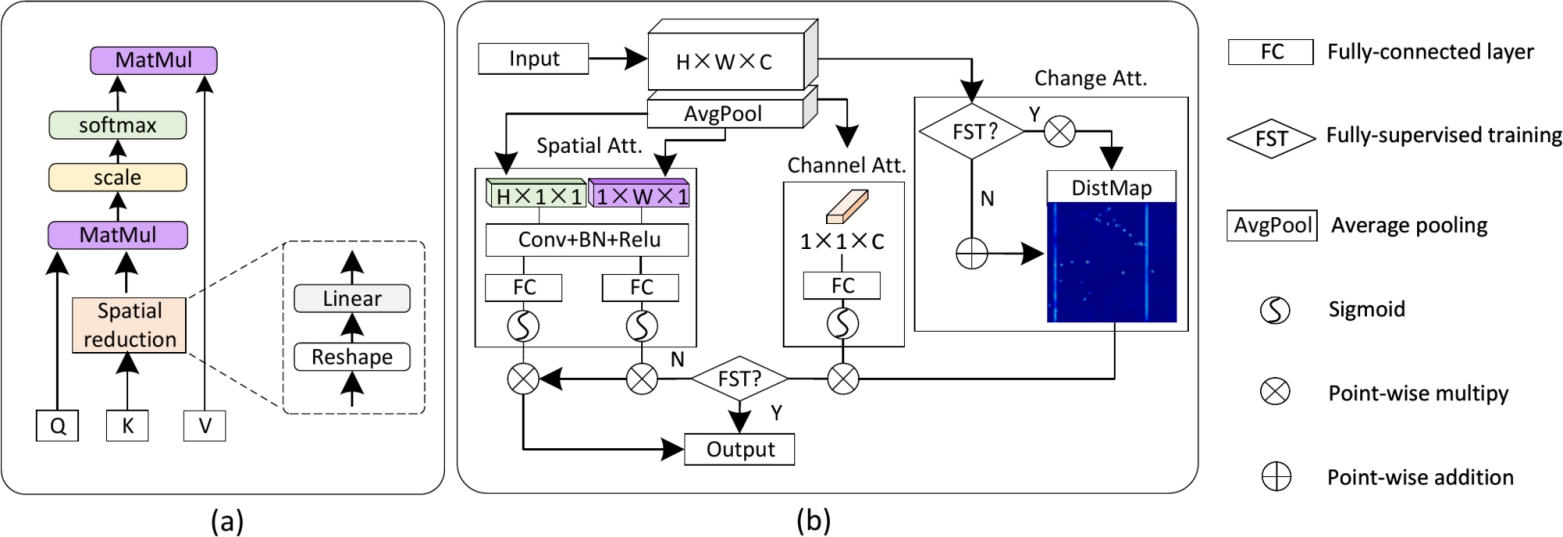
The basic modules. (**a**) The sequence reduction attention utilizes the spatial reduction layer to reduce the complexity of the self-attention module from $O(N^2)$ to $O\left( \frac{N^2}{R}\right)$. (**b**) The change-aware decoder, based on a 3-dimensional (horizontal, vertical, and depth) attention module, utilizes the DistMap carrying change information in different ways when detecting objects in fully-supervised and semi-supervised settings.

We designed an efficient Transformer-based encoder to learn contrastive features with an implicit metric for feature comparison, ensuring it meets the demands of fast inspection in industrial production. To improve the efficiency since there are double computation costs for processing paired inputs, we draw the inspiration **from** sequence reduction attention^29,60^, as illustrated in Fig. 3 (a). A major bottleneck of the vanilla self-attention mechanism^59^ is the quadratic complexity with long sequence inputs, which is:

4 $$\begin{aligned} \text {Attention}(Q,K,V) = \text {Softmax}(\frac{QK^T}{\sqrt{d_{k}}})V, \end{aligned}$$

where matrices *Q*, *K*,  and *V* have the same dimensions $N\times C$, and $d_k=N$. We adopt the ratio *R* to reduce the length of sequence *K* as follows:

5 $$\begin{aligned} & {\hat{K}} = \text {reshape}(\frac{N}{R},C\cdot R)(K), \end{aligned}$$

6 $$\begin{aligned} & K = \textrm{linear}(C\cdot R, C)({\hat{K}}), \end{aligned}$$

where the sequence *K* is initially reshaped to $\frac{N}{R} \times C \cdot R$, followed by a linear layer that processes a sequence of length $(C \cdot R)$ and produces a *C*-dimensional sequence. Consequently, the dimensions of the new *K* become $\frac{N}{R} \times C$, effectively reducing the complexity of the self-attention process from $O(N^2)$ to $O\left( \frac{N^2}{R}\right)$. Each sequence reduction attention (SRA) module comprises a residually connected sequence reduction attention unit and a multi-layer perceptron (MLP). We employ two SRA modules at each Transformer stage, assigning reduction ratios of $\{ 8, 4, 2, 1\}$ for the four stages, respectively.

The hierarchical Transformer blocks encode the defective and defect-free images in parallel using shared weights since the image pairs differ only in minimal defective regions. Denoting the pyramid features as $\{f_m^n|m={0,1},n={0,1,2,3}\}$, where *m* indicates the two Siamese branches, and *n* denotes the four feature layers. The feature distance at position (*i*, *j*) is:

7 $$\begin{aligned} \begin{aligned} \text {DistMap}\left( i,j\right)&= \left\| f ^\text {ng}\left( i,j\right) -f ^\text {ok}\left( i,j\right) \right\| _{2},\\ f ^\text {ng}&=\textrm{concat}{({f_0^1,f_0^2,f_0^3,f_0^4})},\\ f ^\text {ok}&=\textrm{concat}{({f_1^1,f_1^2,f_1^3,f_1^4})}, \end{aligned} \end{aligned}$$

where $f^{ng}$ and $f^{ok}$ denote the concatenated (concat) hierarchical features from defective and defect-free images, respectively. The hierarchical features are resized to match the size of $F^1_0$ or $F^1_1$. The contrastive loss (CL) is adopted as a constraint, which is formulated as:

8 $$\begin{aligned} \text {CL}=\left\{ \begin{array}{cc} \text {DistMap}\left( i,j \right) -{\tau }_{ok},\quad \quad & y(i,j)=0, \\ \max \left( 0,{\tau }_\text {ng}-\text {DistMap}\left( i,j \right) \right) ,\quad & y(i,j)=1, \\ \end{array}\right. \end{aligned}$$

where *y*(*i*, *j*) is the ground truth, with values 0 or 1 indicating whether the point is unchanged or changed, respectively. ${\tau }_\text {ok}$ and ${\tau }_\text {ng}$ are non-negative thresholds. When $y(i,j)=0$ (*i.e.*, unchanged point), the feature distance is expected to reduce towards ${\tau }_\text {ok}$, which is close to 0. Conversely, when $y(i,j)=1$ (*i.e.*, changed point), the feature distance is encouraged to increase towards ${\tau }_\text {ng}$. We set the ${\tau }_\text {ng}$ and ${\tau }_\text {ok}$ as 2.2 and 0.3 according to^61^. The ${\tau }_\text {ok}$ is a positive value slightly above 0 since the paired unchanged points (defect-free) are not exactly the same. The ${\tau }_\text {ng}$ is set to a larger positive value to encourage a considerable margin between these points that are different or defective.

The original contrastive loss is proposed for binary change detection. However, when there is more than one type of defect to be modeled as changed regions (*i.e.*, $y \in {1,2,..,c}$), the sample-amount imbalance between them leads to imbalanced contrastive supervision. Hence, we propose to extend it with a multi-class balanced factor. Given the proportion of certain change categories to the total change areas (*i.e.*, $y(i,j)=1$), the balance factor is defined as:

9 $$\begin{aligned} B_{p}=\frac{1}{{f}_{p}}=\frac{1}{{{n}_{p}}}{\sum \limits _{q}^{C}{{{n}_{q}}}}. \end{aligned}$$

where C is the number of total classes, ${f}_{q}$ is the ratio of class *q* sample points to the total number of change sample points, where $n_q$ and $n_p$ denote the number of points in class *q* and class *p*, respectively. The balanced contrastive loss (BCL) can be defined as:

10 $$\begin{aligned} \text {BCL}=\left\{ \begin{array}{ccc} & \text {CL}, & \ y(i,j)=0, \\ & \sum \limits _{{c}^{l}=0}^{C}{{B}_{l}}, \cdot \text {CL}({y(i,j)}=c^l) & \ y(i,j)\in {1,2,..,C}. \end{array}\right. \end{aligned}$$

It places greater emphasis on less common change categories, resulting in a well-balanced distribution of loss across different types of changes.

#### Change-aware decoder

The attention mechanism is widely applied to model contextual information. However, the arbitrary location distribution and weak association with the surroundings of defects seriously affect the spatial context. To this end, we proposed a novel change attention mechanism named change-aware decoder (CAD), which introduces change information to assist in the location of the defect objects. Specifically, the feature distance obtained from the contrastive feature encoder is skip-connected to the decoder and plays distinct roles when detecting objects in fully-supervised and semi-supervised settings. The structure of CAD is shown in Fig. 3 (b).

Initially, we extend the lightweight coordAttention^62^ into a 3-dimensional attention module, which allows us to achieve considerable precision in feature decoding while maintaining a low parameter cost. Constrained by the balanced contrastive loss, the DistMap exhibits high activation values for the change region and low values for the constant region. Current semantic segmentation methods have proven effective when detecting defects with abundant labeled data. Hence, the feature distance is added to the encoded features to assist in locating defects, which are represented as:

11 $$\begin{aligned} \text {Output}=\operatorname {ChangeAtt}(\text {Input} \oplus \text {DistMap}), \end{aligned}$$

where $\oplus$ means point-wise addition, and $\operatorname {ChangeAtt}$ here is the combination of channel Attention (CA), horizontal attention (HA), and vertical attention (VA). The $\operatorname {ChangeAtt}$ is derived from:

12 $$\begin{aligned} \operatorname {ChangeAtt}( \cdot ) = \text {CA}( \cdot ) \otimes \text {HA}( \cdot )\otimes \text {VA}( \cdot ), \end{aligned}$$

where $\otimes$ means point-wise multiplication. However, when encountering unseen defects with unknown appearances due to the limitation of labeled data, reliance on defect appearance becomes unreliable. In fact, it could be argued that when defect patterns are overfitted on the training set, it may lead to poorer generalization performance on the test set. In such scenarios, change information becomes the primary indicator for defect localization. Consequently, the DistMap acts as the spatial **cue** and interacts with the encoded features multiplicatively after normalization ($\operatorname {Norm}$) to aid in this process, which is

13 $$\begin{aligned} & \operatorname {ChangeAtt}( \cdot ) =\text {CA}( \cdot ) \otimes \operatorname {Norm}(\text {DistMap})\otimes ( \cdot ), \end{aligned}$$

14 $$\begin{aligned} & \text {Output}=\operatorname {ChangeAtt}(\text {Input}). \end{aligned}$$

In this context, the multiplication operation incorporates a robust prior to specifically target the change regions. The DistMap serves as a spatial context prior, replacing the conventional horizontal or vertical attention mechanisms. Its purpose is to guide the model in identifying potential defects within the change areas. Notably, Fig. 7 demonstrates that the DistMap provides a coarse representation of the final outcome, with the so-called defective regions aligning precisely with the actual regions of change.

#### Loss function

The BCL and cross-entropy loss are employed for training the network. The BCL guides the model to learn contrastive features as mentioned in section Contrastive feature encoder. The cross-entropy loss for a single point (*i*, *j*) is defined as:

15 $$\begin{aligned} \text {CEL}=-\log \frac{{{e}^{{\hat{y}}(i,j,{{c}^{y}})}}}{\underset{c^{k}=0}{\overset{C-1}{\mathop {\sum }}}\,{{e}^{{\hat{y}}(i,j,c^{k})}}}, \end{aligned}$$

where ${c}^{y}$ is the true category of a sample point, *C* is the total categories, and ${\hat{y}}(i,j,c^k)$ indicates the predicted probability of class $c^k$. The overall loss function used during model training is as follows:

16 $$\begin{aligned} \textrm{loss}=\lambda _{1} \text {CEL}+\lambda _{2} \text {BCL} \end{aligned}$$

$\lambda _1$ and $\lambda _2$ are set to 1 in our experiment.

## Experiments and results

### Datasets

Three datasets are involved in the evaluation, including our synthetic LCD dataset and the PKU-Market-PCB^31^ dataset, which are characterized by complex backgrounds and tiny texture anomalies. Additionally, the anomaly detection benchmark MVtec-AD^16^ is used to validate the generalizability of our method in a fully-supervised learning setting.

**Fig. 4:**
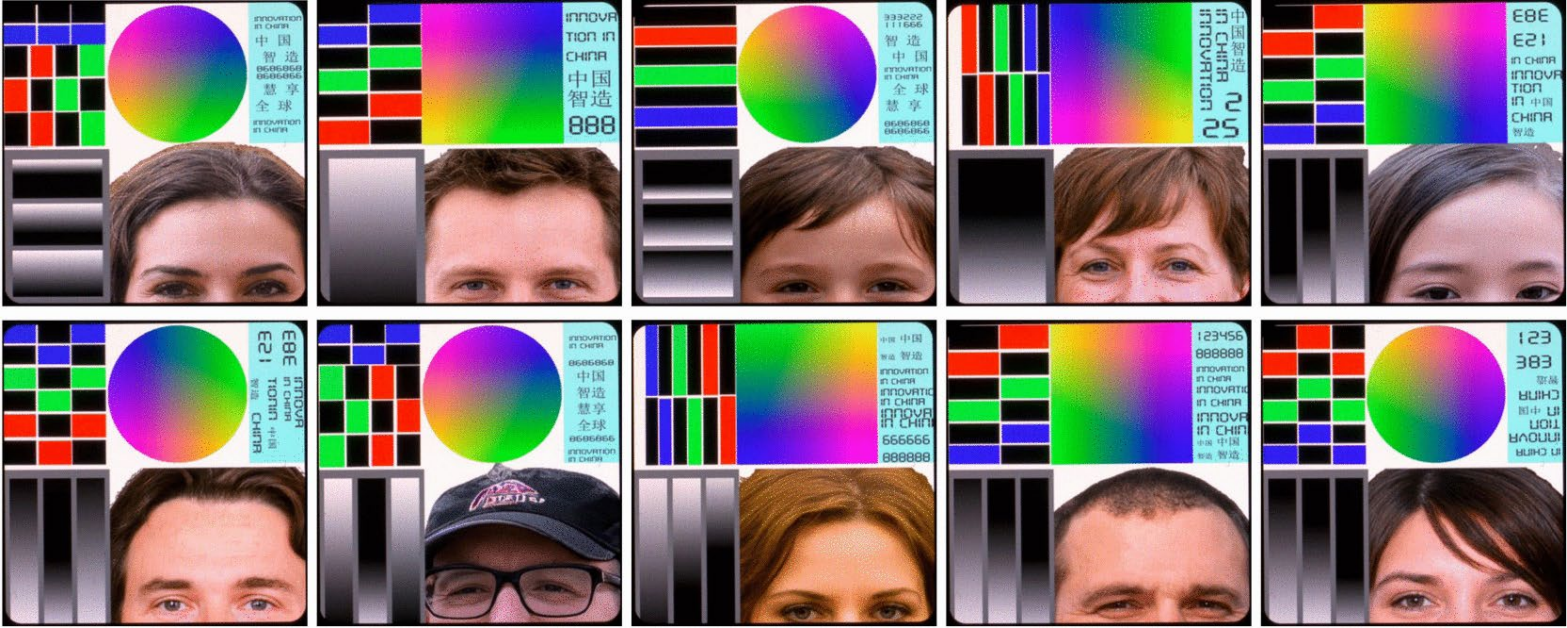
The defect-free LCD patterns. In the real inspection process, the industrial LCD display patterns are constructed with RGB blocks, gray transition, color maps, characters, and faces to reveal various types of defects (*e.g.*, point, line, and Mura defects^63^).

**Synthetic LCD defect dataset**. To validate our model’s capability for segmenting defects under various imaging, production conditions, and defect appearances, we constructed a synthetic LCD defect dataset termed SynLCD. During the real-world LCD inspection process, some specific display patterns are designed to reveal various types of defects (*e.g.*, point, line, and Mura defects^63^). These patterns are constructed with pure color blocks, color maps, text blocks, grayscale transitions, and human faces. Figure 4 depicts ten defect-free display patterns, where the defect detection interface is divided into five functional zones to comprehensively evaluate display performance:

- Bottom-right corner (Facial image): The human visual system is highly sensitive to facial features. This zone tests the display’s ability to accurately render complex biological details, such as skin tone, hair texture, and facial contours. Deviations in color balance, saturation, or texture clarity may indicate panel driver issues or color calibration defects.
- Bottom-left corner (Grayscale transition): This zone uses a gradient from pure black to white to assess brightness uniformity, contrast response, and backlight consistency. Defective screens may exhibit banding (unnatural color steps), uneven brightness, or backlight leakage (e.g., “Mura defects”), compromising visual smoothness.
- Top-right corner (Text patterns): Designed to evaluate high-frequency detail rendering. Low-quality panels often display text with jagged edges, missing pixel blocks, or background noise-issues linked to abnormal subpixel arrangements, unstable driving circuits, or TFT (thin-film transistor) array defects.
- Above the facial image (RGB chromaticity chart): Utilizes standardized color wheels or gradients to measure color gamut coverage and accuracy. Abnormalities like color shifts, aliasing, or desaturation suggest flaws in color filters or signal processing algorithms.
- Top-left corner (Solid color blocks): Includes primary colors (red, green, blue, black) to test color purity and backlight uniformity. Irregularities such as localized brightness fluctuations, color blotches, or dark spots (e.g., “line defects”) reveal manufacturing imperfections.

**Fig. 5:**
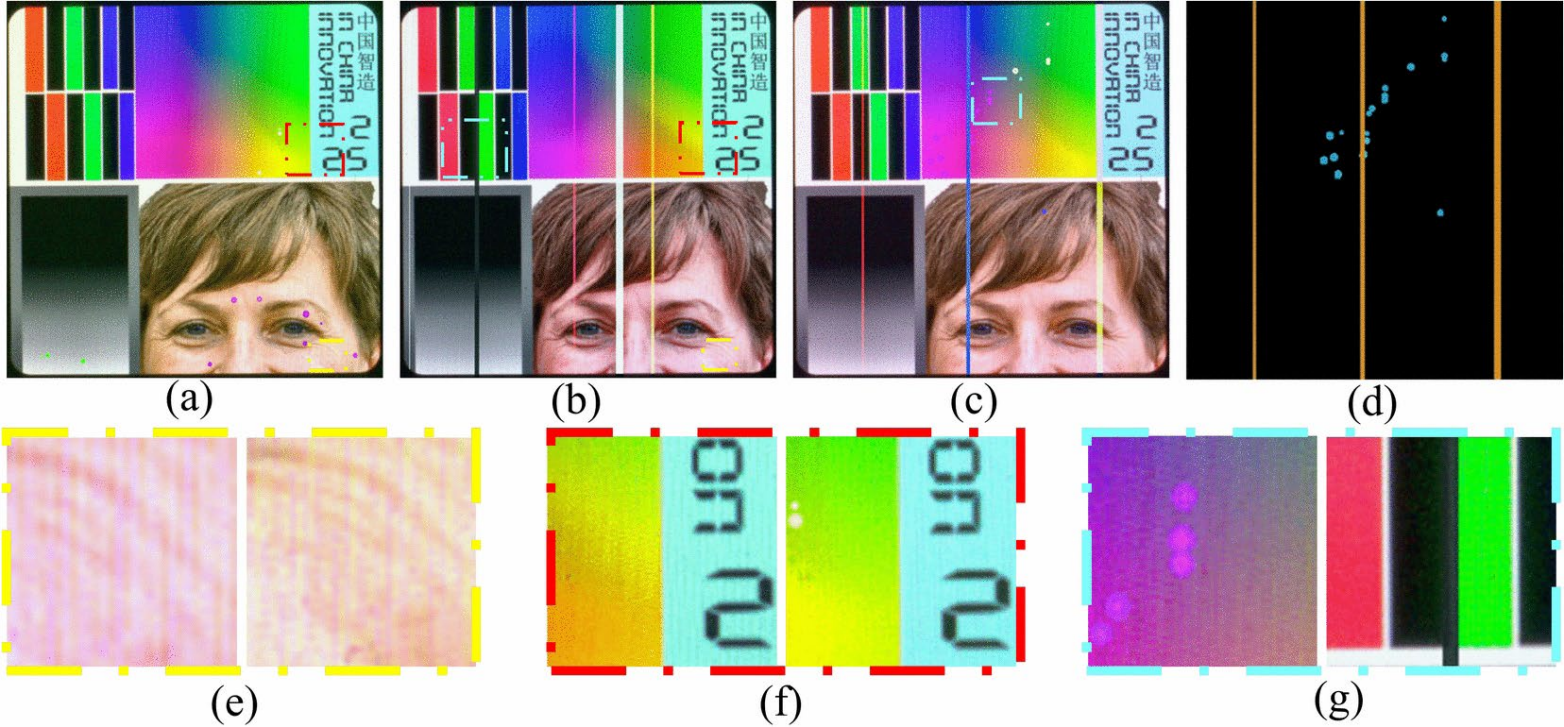
Samples of SynLCD and the dataset challenges. (**a**) Abnormal points defect sample; (**b**) line defect sample; (**c**) mixed defect sample; (**d**) binary label of mixed defect image. (**e**) RGB deviation and irregular screen texture; (**f**) nonlinear saturation difference. (**g**) low contrast abpt and line defects.

The SynLCD dataset includes three types of defect samples with random positions and distribution: line defects, abnormal points (abpt), and mixed defects, as presented in Fig. 5. Some of these defects closely resemble the background patterns. For line defects, they exhibit low contrast with the background, spanning across the entire screen.

**Table 1:** Statistical details of SynLCD dataset.

Attributes	Type	Values	Remarks
Amount	background pattern	10	variation in face and color-map, etc.
	Defect types	3	line, abpt and mixed defects
	Defect Samples	10$\times$300$\times$3	300 samples for each type and each pattern
	Nondefect Samples	10$\times$900	variation in brightness and contrast, etc.
Defect	shape	2	line and abpt
	color	5	black, white, red, green, blue
	opacity	10$\%$-100$\%$	10$\%$ interval
	width	3-33 pixels	3$\%$ interval
Screen	brightness	bias: 1-6	1 interval
	contrast	alpha: 0.5-1.5	0.1 interval
	ISO noise	10$\%$-100$\%$	10$\%$ interval
	RGB deviation	3-33 grayscale	3 interval

Table 1 shows the statistical details of SynLCD. According to the assumption in Eq. (1), the defect image $x_\text {ng}$ is formed by superimposing a clean surface image $x_\text {ok}$ with the defect $x_\text {defect}$ after applying a non-linear overall surface change $\sigma$. To generate line defects, we first divide the clean image into **K** areas. Next, in each area, we pre-draw a line with random color, transparency, and width. These lines traverse the screen, simulating real-world line defects. Abnormal points tend to appear in high-frequency transition regions such as edges, hair, and text. To create abpt samples, we vary the grayscale threshold from 50 to 200 to obtain segmentation results at each threshold. From these segmentation results, we extract a set of edge points. Subsequently, we randomly cluster these points using K-means clustering, assigning each subclass a random color, scale, and transparency. Once we obtain both types of defects, we overlay them onto the clean image using Gaussian blur and Poisson seamless fusion^64^. This process introduces random luminance, contrast, ISO noise, and RGB color bias, enhancing sample diversity. To prevent sample imbalance interference during the classification task, we generate 300 defective and defect-free samples for each clean image in Fig. 4. In total, there are 4,200 training samples (seven standard background patterns and 600 samples for each pattern) and 1,800 testing samples.

**PKU-Market-PCB**. The PKU-Market-PCB dataset^31^ comprises 1,386 images along with 6 types of defects to validate the generalizability of our model in scenes with complex background and tiny defects. The original images exhibit inconsistent sizes. To streamline the training process, we resized and cropped the original images into $1000\times 1000$ sub-images, retaining only those containing defects. Finally, there are 1,566 (70%) images for training and 676 (30%) images for testing. The preprocessed PCB dataset is included with our source code for accessibility at https://github.com/HATFormer/CADNet.

**MvTec-AD.** To further validate our model in detecting general defects, we conduct a comparison using the MvTec-AD^16^. It is a widely used anomaly detection benchmark. To facilitate more effective training and achieve precise defect segmentation, we reorganized the original dataset for fully-supervised training. The original 5,354 images, along with their corresponding ground-truth annotations, were randomly shuffled and divided into two subsets: 3,747 (70%) images for training and 1,607 (30%) images for testing.

### Experiment setting and metrics

**Implementation details**. Our model is implemented with MMSegmentation and trained with an RTX3090 GPU. The input images are resized into $512\times 512$ with common data augmentations, including random crop, flip, and color normalizing during training. For each dataset in the case of fully-supervised comparison, 70% and 30% of samples are used for training and testing, respectively. For the compared methods, the input consists of a sample from the original dataset. In contrast, our method takes both a sample from the original dataset and the corresponding clean image as inputs. For SynLCD, there are ten clean fixed images, as shown in Fig. 4. Similarly, for PKU-Market-PCB^31^, ten clean images are provided. For MvTec-AD^16^, one defect-free image per class is selected for both training and testing. All models are trained for 30 epochs with a mixed batch size ranging from 4 to 8, depending on the memory usage of each specific model.

In the context of semi-supervised comparison, we vary the proportion of labeled samples between 0% (This is actually an unsupervised setting. For simplicity, we include it in the semi-supervised comparison), 5%, 10%, and 15%. To compare with UAPS^5^, which utilizes unlabeled data for training, we follow the established setting in^5^ by incorporating 10% of unlabeled data.

**Metrics**. We use the semantic segmentation metrics for evaluating the pixel-wise defect predictions, including mean Intersection over Union (mIoU), Accuracy (Acc), and Fscore as also denoted in^36,65^. TP, FP, and FN are abbreviations for True Positive, False Positive, and False Negative, respectively. The metrics are outlined as follows:

- precision (P) and recall (R): ${ \text {TP/(TP+FP)}, \text {TP/(TP+FN)}}$,
- Fscore: $$\text {2PR/(P+R)}$$,
- accuracy (Acc): $${\text {TP}+\text {TN}}/(\text {TP}+\text {FN}+\text {FP}+\text {TN})$$,
- mIoU: $$\frac{1}{C} \sum _{i=0}^{(C-1)} \frac{\text {TP}_i}{\text {TP}_i+\text {FP}_i+\text {FN}_i}$$.

We measured the model’s complexity using parameters (Params) and giga floating point operations (GFLOPs). In all tables, the up-arrow means the higher the better, while the down-arrow means the lower the better.

**Compared methods**. Our model is evaluated from two aspects: (1) The fully-supervised segmentation task aims to demonstrate the superiority of change modeling over appearance modeling when there are abundant pixel-wise labels available. Six semantic segmentation methods are involved for comparison, as shown in Table 2. (2) The semi-supervised segmentation aims to evaluate the model robustness facing insufficient labeled samples as defects in a real-world production environment would not have abundant samples with consistent defect appearance. Five SOTA semi-supervised methods are involved for comparison as given in Table 2.

**Table 2:** An overview of fully-supervised and semi-supervised segmentation methods for comparison.

Fully-Supervised Methods	Semi-Supervised Methods
**FCN**^66^: utilizes fully convolutional layers to realize dense prediction for arbitrary-sized images.	**DCT**^45^: employs one network to ensure consistency across different views of a given sample.
**PSPNet**^67^: Utilizes global context aggregation through pyramid pooling for complicated scene parsing.	**CPS**^43^: enforces consistency between two segmentation networks initialized differently.
**DeepLabV3+**^36^: introduced the atrous spatial convolutional pyramid (ASPP) to enhance the multi-scale contextual information.	**UAMT**^46^: encourages consistent predictions under different perturbations and estimates uncertainty to learn from unlabeled data.
**DANet**^68^: enhances segmentation by adaptively integrating semantic dependencies in spatial and channel dimensions via the self-attention mechanism.	**UCC**^44^: employs a shared encoder with dual decoders and enforces consistency between the decoders with data augmentations.
**OCRNet**^69^: introduces object-contextual representations for semantic segmentation, leveraging pixel-object relationships to augment pixel representations.	**UAPS**^5^: dynamically blends pseudo-labels from multi-head outputs during a single forward pass for uncertainty regularization.
**SegFormer**^40^: presents a streamlined semantic segmentation framework by integrating Transformers with lightweight MLP decoders.	

### Quantitative comparison

#### Fully-supervised segmentation

In this section, we evaluate our proposed method in terms of fully-supervised segmentation performance. From the results of Table 3, our model achieves a remarkable improvement over the other segmentation models. Specifically, our model exhibits improved performance across the four metrics ($\mathrm {IoU_{line}}$, $\mathrm {IoU_{abpt}}$, $\textrm{mIoU}$, $\textrm{mFscore}$) by 12.65%, 0.82%, 8.17%, and 4.15%, compared to the runner-up results. In Table 4 and 5, our model obtains the best outcomes across most metrics in the PCB and MvTec-AD datasets.

**Table 3:** Comparison with the mainstream semantic segmentation methods in SynLCD dataset. **Bold**, ***Bolditalic*** and *italic* indicate the top three results for each metric.

Method	$IOU_\text {line}\uparrow$	$\hbox {IOU}_\text {abpt}\uparrow$	$\hbox {mIOU}\uparrow$	$\hbox {mAcc}\uparrow$	$\hbox {mFscore}\uparrow$	$\hbox {MParams}\downarrow$	$\hbox {GFLOPs}\downarrow$
FCN^66^	51.86	11.48	31.67	36.06	44.45	49.5	57.91
PSPNet^67^	79.00	52.54	65.77	71.56	78.58	12.76	54.27
DeepLabV3+^36^	81.96	***72.93***	***77.45***	**90.24**	***87.22***	43.58	176.22
DANet^68^	79.92	57.04	68.48	76.27	80.74	49.82	199.05
OCRNet^69^	***83.46***	62.19	72.83	*86.08*	83.84	12.07	52.83
SegFormer^40^	*82.99*	*69.62*	*76.31*	83.68	*86.39*	3.72	6.37
Our-CADNet	**94.02**	**73.53**	**83.78**	***89.05***	**90.84**	3.90	8.21

**Table 4:** Comparison with the mainstream semantic segmentation methods in the PCB Dataset. **Bold**, ***Bolditalic*** and *italic* indicate the top three results for each metric.

Method	$\hbox {IOU}_{c1}\uparrow$	$\hbox {IOU}_{c2}\uparrow$	$\hbox {IOU}_{c3}\uparrow$	$\hbox {IOU}_{c4}\uparrow$	$\hbox {IOU}_{c5}\uparrow$	$\hbox {IOU}_{c6}\uparrow$	$\hbox {mIOU}\uparrow$	$\hbox {mAcc}\uparrow$	$\hbox {mFscore}\uparrow$
FCN^66^	50.13	69.19	68.65	45.45	50.35	36.36	53.35	60.80	68.79
PSPNet^67^	74.04	72.59	72.61	71.29	66.39	72.46	71.56	81.77	83.40
DeepLabV3+^36^	75.39	***73.56***	***74.22***	*73.57*	69.94	76.47	*73.85*	82.10	*84.94*
DANet^68^	74.31	*73.02*	71.21	72.14	68.86	75.02	72.42	82.01	83.99
OCRNet^69^	***76.08***	73.00	*73.78*	***75.98***	***71.13***	***78.13***	***74.68***	***83.45***	***85.48***
SegFormer^40^	*75.79*	71.39	72.31	72.29	*70.75*	*78.04*	73.42	*82.29*	84.65
Our-CADNet	** 77.21**	** 73.98**	** 75.08**	** 79.95**	** 76.47**	** 82.44**	** 77.52**	** 85.87**	** 87.31**

In terms of efficiency, our model has comparable parameter to SegFormer, and both surpass other models significantly in computation. Our model shows substantial improvements over SegFormer, with a 1.84 GFLOPs increase resulting in 9.79% higher mIOU, 6.42% higher mAcc, and 5.15% higher mFscore. On one hand, this highlights our model’s efficiency, making it well-suited for deployment in industrial devices with limited computational resources. On the other hand, it showcases the superiority of our change-modeling mechanism.

**Table 5:** Comparison with the mainstream semantic segmentation methods in the MvTec-AD Dataset. **Bold**, ***Bolditalic*** and *italic* indicate the top three results. Note that there are 15 classes in MvTec-AD and six of them are reported here.

Method	$\hbox {IOU}_{c1}\uparrow$	$\hbox {IOU}_{c2}\uparrow$	$\hbox {IOU}_{c3}\uparrow$	$\hbox {IOU}_{c4}\uparrow$	$\hbox {IOU}_{c5} \uparrow$	$\hbox {IOU}_{c6}\uparrow$	$\hbox {mIOU}\uparrow$	$\hbox {mAcc}\uparrow$	$\hbox {mFscore}\uparrow$
FCN^66^	76.10	60.14	35.93	69.73	13.51	79.65	58.14	64.84	70.00
PSPNet^67^	72.00	***68.24***	43.86	**74.89**	*42.43*	*83.44*	*65.42*	*76.25*	***77.58***
DeepLabV3+^36^	*76.65*	63.48	41.18	72.31	34.93	81.12	63.77	***77.59***	76.19
DANet^68^	75.13	56.37	37.95	*72.42*	27.10	80.92	61.63	72.49	73.94
OCRNet^69^	70.89	*65.18*	*45.67*	65.47	35.41	81.51	59.89	68.98	72.31
SegFormer^40^	***81.63***	64.63	***53.81***	70.81	***44.14***	***84.71***	***65.97***	71.21	*77.51*
Our-CADNet	**82.60**	**74.16**	**61.19**	***73.06***	**52.69**	**86.41**	**71.35**	**80.85**	**82.24**

#### Semi-supervised segmentation

When defect appearances are clearly defined with ample labeled data, general segmentation models like SegFormer demonstrate satisfactory performance. However, a notable concern is that appearance-based modeling cannot ensure robust generalization in real-world applications with the limited defect samples and diverse defect appearance. Therefore, we explore defect segmentation more deeply in scenarios with limited or even no labels.

**Fig. 6:**
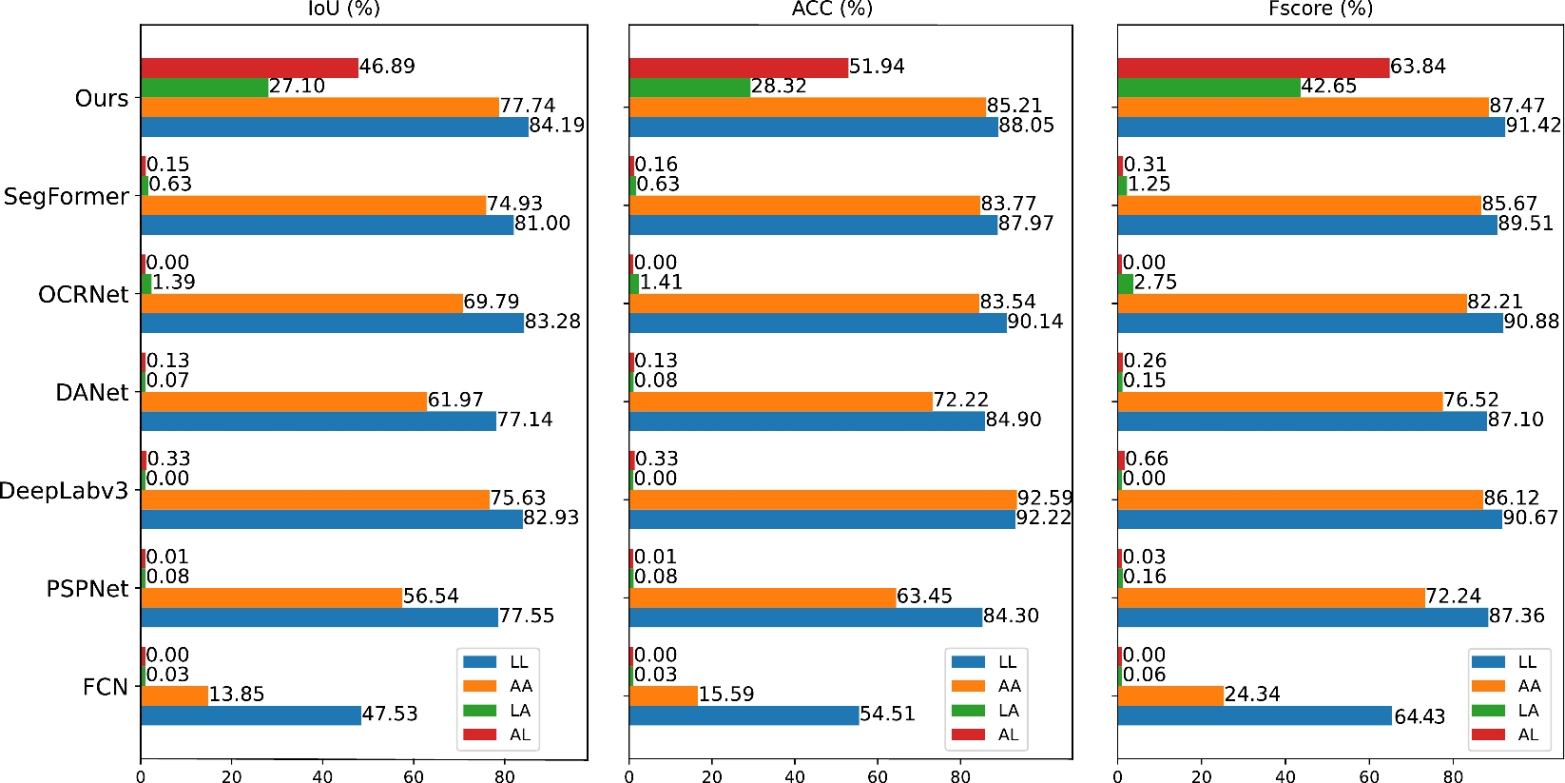
Comparison of cross-testing performance. In this setting, the samples during inference do not appear in the training phase. For LL, AA, LA, and AL, the first character means training with line (L) or abpt (A) set, while the second represents the testing set.

In the series of experiments, denoted as LL, AA, LA, and AL, the first character indicates training on either line (L) or abpt (A), while the second character denotes testing on line (L) or abpt (A). As results shown in Fig. 6, most segmentation models obtain acceptable fully-supervised segmentation results but fail to detect unseen defects (metrics such as IoU, Acc, and Fscore are lower than 0.5%) due to their appearance-based modeling nature. In contrast, our change-aware model exhibits considerable results when defect appearance is unseen in the training phase. Regarding LA (*i.e.*, trained online and tested on abpt defect), there is a notable decrease in accuracy. It is conceivable that the abpt defects are more complex to distinguish from the background with smaller sizes.

**Table 6:** Comparison with the SOTA semi-supervised segmentation methods in the SynLCD dataset across varying proportions of labeled data (from 0% to 15%). All models are pre-trained on the abpt defects and subsequently fine-tuned and tested using the line defects. The bold font indicates the best results.

Method	$\hbox {mIoU}\uparrow$				$\hbox {Fscore}\uparrow$			
	0%	5%	10%	15%	0%	5%	10%	15%
DCT^45^	0.05	56.96	73.67	71.85	0.10	71.27	84.57	82.75
UAMT^46^	0.44	61.68	68.73	71.96	0.88	75.48	80.94	83.15
CPS^43^	1.09	65.07	65.63	76.02	2.15	78.29	78.70	85.68
UCC^44^	0.015	61.40	70.48	71.55	0.03	75.41	82.27	82.78
UAPS^5^	0.44	58.86	74.43	81.34	0.88	72.52	84.35	89.22
Our-CADNet	**46.89**	**82.93**	**84.52**	**84.71**	**63.84**	**90.87**	**91.64**	**91.72**

Table 6 demonstrates our model’s superior performance to five SOTA semi-supervised segmentation methods across different supervision settings. This demonstrates our model’s potential for flexible application with only a few real samples in production, significantly reducing data collection and labeling costs.

### Ablation studies

**Fig. 7:**
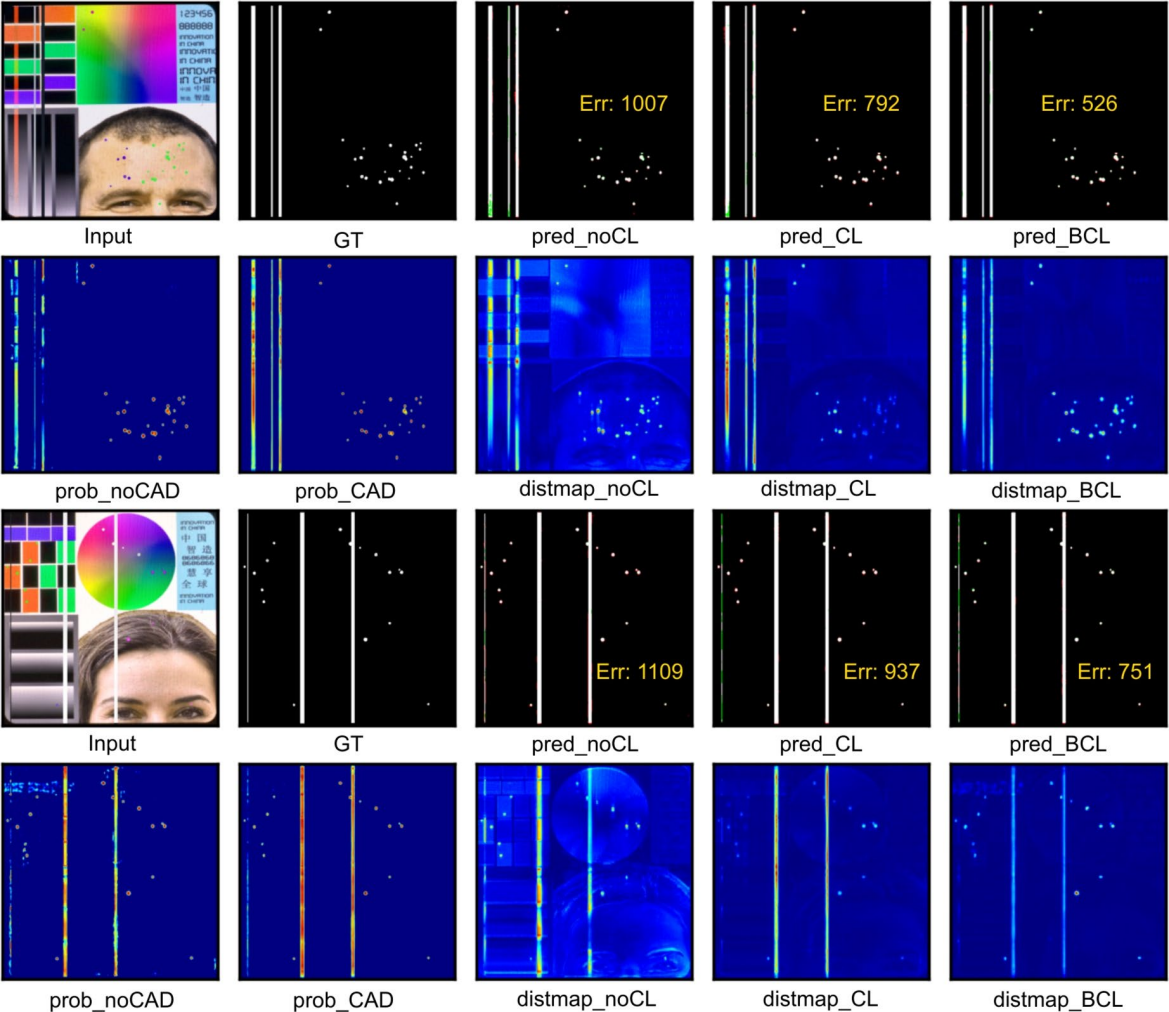
Visual ablation results. It shows the final predictions (pred), probability map (prob) before output and DistMap with or without CAD, CL, and BCL.

In this section, we investigate how the contrastive loss (CL), balanced contrastive loss (BCL), and change-aware decoder (CAD) influence the model. According to the results in Table 7 and Fig. 7, the following conclusions can be drawn:

**Table 7:** Ablation study about the loss function and decoder. From left to right are cross-entropy loss (CEL), contrastive loss (CL), balanced contrastive loss (BCL), and change-aware decoder.

**CEL**	**CL**	**BCL**	**CAD**	$\hbox {IoU}_{line}\uparrow$	$\hbox {IoU}_{abpt}\uparrow$	$\hbox {mIoU}\uparrow$	$\hbox {mAcc}\uparrow$	$\hbox {mFscore}\uparrow$	$\hbox {Params}\downarrow$	$\hbox {GFLOPs}\downarrow$
$\checkmark$				84.21	73.00	78.61	85.09	87.91	3.72	8.16
$\checkmark$	$\checkmark$			89.40	70.17	79.78	85.22	88.43	3.72	8.16
$\checkmark$		$\checkmark$		89.56	72.96	81.26	87.32	89.43	3.72	8.16
$\checkmark$		$\checkmark$	$\checkmark$	94.02	73.53	83.78	89.05	90.84	3.90	8.21

- Leveraging CL to supervise intermediate layers has led to notable improvements in most accuracy metrics without introducing extra computational costs. Comparison between distmap_noCL and distmap_CL in Fig. 7 highlights how the contrastive constraint aids in reducing background noise and identifying more discriminative change (defective) regions. Furthermore, distmap_noCL illustrates that lines are more discernible than abpt regions, indicating an imbalanced contrastive constraint.
- As depicted in distmap_CL and distmap_BCL in Fig. 7, BCL effectively amplifies the intensity of abpt defects, leading to a further improvement in $\text {IoU}_\text {abpt}$ while maintaining stable $\text {IoU}_\text {line}$. Consequently, there is an overall increase in mIoU and mFscore.
- The CAD model yields enhancements across all accuracy metrics with a minimal increase of computation cost. The comparison between prob_noCAM and prob_CAM reveals the significance of change information and spatial context in effectively restoring broken lines while mitigating noise detections.

### Qualitative results

**Fig. 8:**
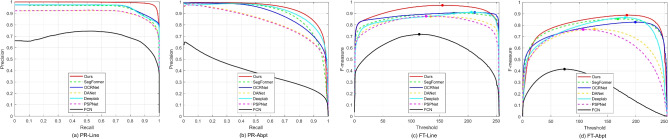
Comparison though precision-recall (PR) and Fscore-threshold (FT) curves. From left to right, the PR curves of the line, the PR curves of the abpt, the FT curves of the line, and the FT curve of the abpt defects.

In the two left panels of Fig. 8, the Precision-Recall (P-R) curves demonstrate that our change-aware network consistently outperforms others, particularly at higher recall values, for both the line and abpt defects. Examining the Fscore-Threshold (FT) curves in the right two panels, our model consistently achieves a higher Fscore across various binary threshold values. Furthermore, the detection of larger-sized line defects generally results in higher precision and Fscore compared to abpt defects.

**Fig. 9:**
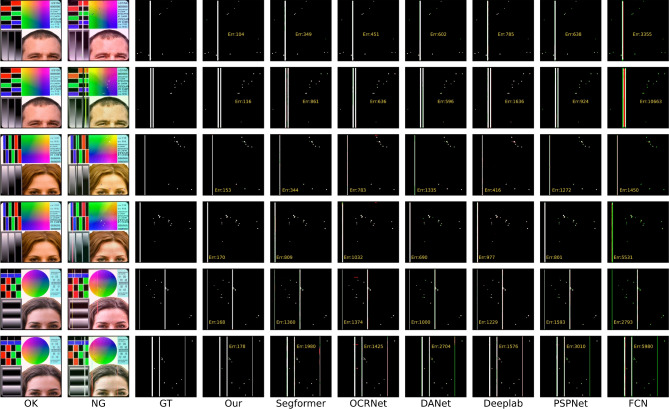
Visual comparison in SynLCD dataset. White color represents the line and abpt defects, while green color represents missed detections and red color wrong detections. The errors (Err) in yellow summarise the missed and wrong detections.

**Fig. 10:**
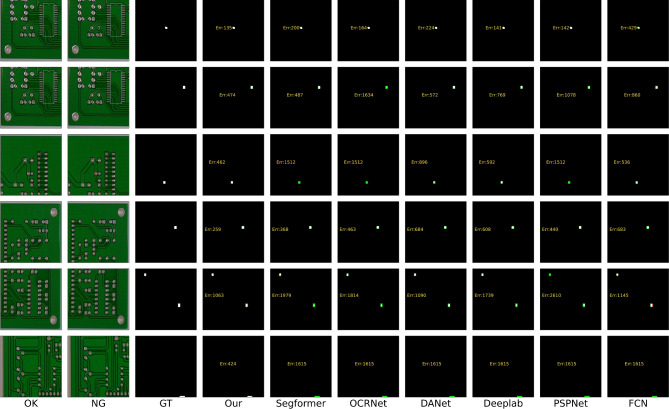
Visual comparison on the PCB dataset. White color represents the defects, while green color represents missed detections and red color wrong detections. The errors (Err) in yellow summarise the missed and wrong detections.

Figs. 9 and 10 present further visual comparisons in the SynLCD and PCB datasets. For an intuitive observation, the multi-class defects are all set to white. The green color denotes missed detections and red color denotes wrong detections. The errors in yellow summarise the missed and wrong detections. Thin lines, in comparison, are more likely to be missed than thick lines, as the downsampling during feature extraction may cause information loss. Overall, the other models are the least effective compared to our CADNet, as reflected by its accuracy metrics. It has a large number of misses and wrong detections on all the tested images.

**Fig. 11:**
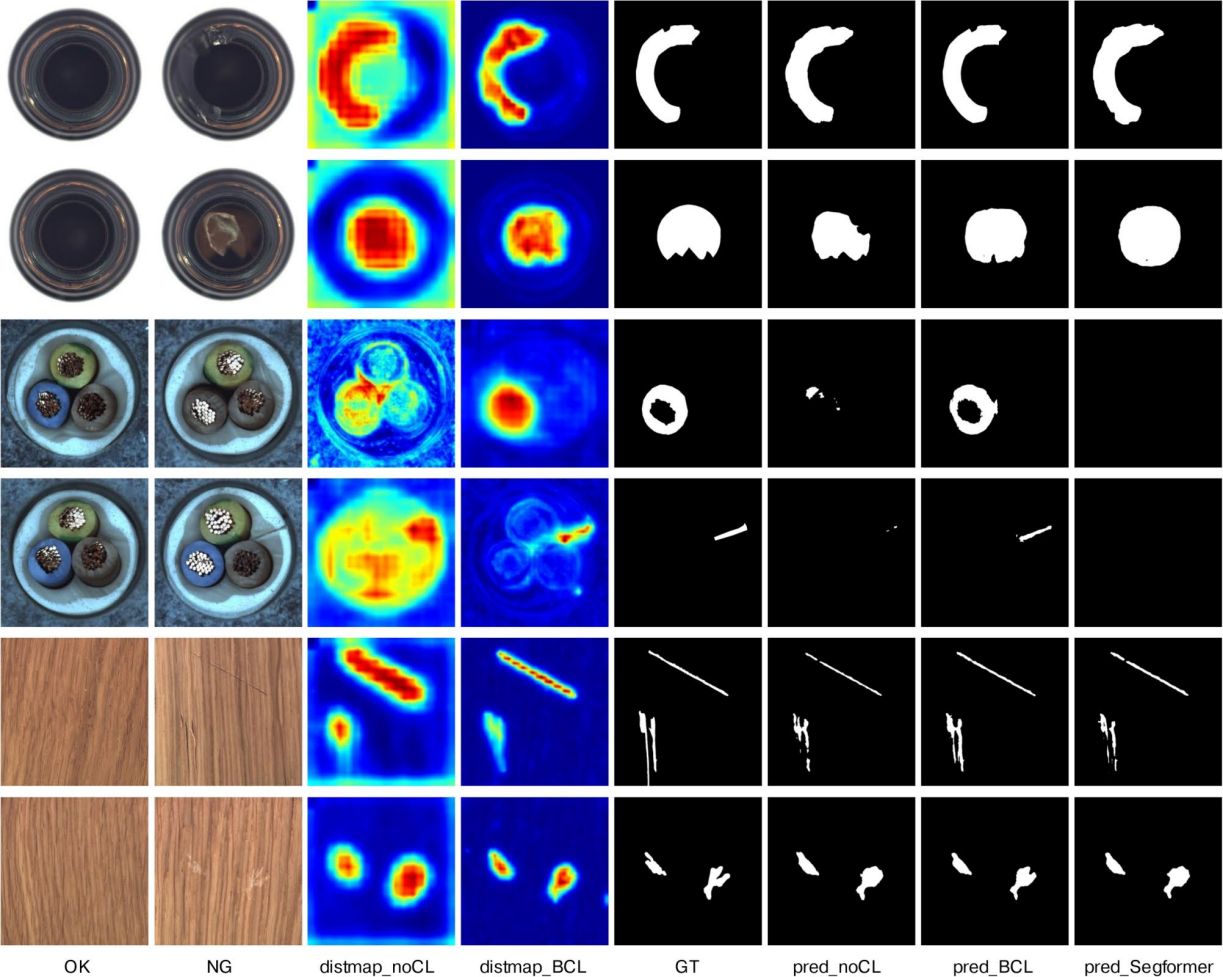
Visual comparison on the MvTec-AD dataset. The results indicate the superior performance of our method with contrastive constraint. The first two and last two rows represent the scenes with low-contrast and complex backgrounds, while the third and fourth rows are high-level semantic anomalies.

**Fig. 12:**
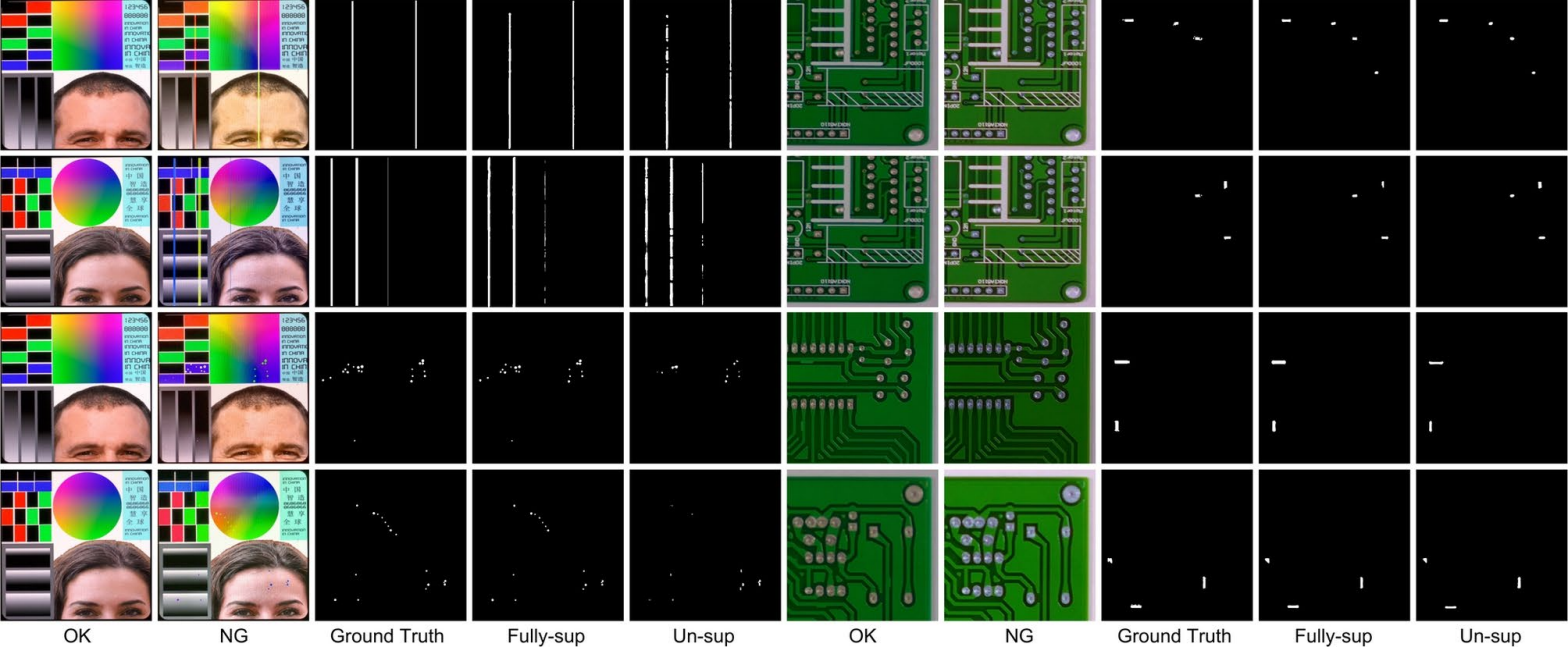
Visualization of model predictions in training settings of fully- and semi-supervision.

Figure 11 depicts the results of our method and Segformer in scenes with general industrial products. Note that the high-level semantic defects in rows 3 and 4 cannot be addressed using conventional segmentation methods, as they exhibit normal textures. Figure 12 illustrates the predictions generated by our model in fully- and unsupervised manners. Interestingly, despite the decline in the mIoU in detecting unseen defects, the visual impact is not readily apparent. Indeed, the mIoU values for CADNet remain impressively satisfactory. Taking the results of SegFormer on the COCO^70^ dataset as benchmarks, the real-time variant of SegFormer (B0) achieves mIoU scores of 35.6%. The non-real-time version (B5) achieves 46.7%. This explains why our method achieves acceptable visual results on unseen objects.

## Conclusion

Recent advancements in computer vision technologies have catalyzed significant progress in industrial defect detection. However, challenges persist in achieving fine-grained defect segmentation, primarily due to two major obstacles: (1) The limited collection and labeling of defect data, which renders most data-driven semantic segmentation models ineffective, and (2) The inconsistent appearances observed during the development and application phases of models, presenting significant bottlenecks to many appearance-modeling algorithms, especially for those defect objects under complex background, including LCD screen, PCB, and printed productions. To address this, we develop a change-based modeling framework to locate pixel-wise multi-class defects, based on the assumption that defective regions are essentially the differences between defective and defect-free images.

We conducted an in-depth experiment using the proposed SynLCD and two public datasets. Our model surpasses six leading segmentation models in performance while maintaining reasonable computational costs. Furthermore, our model demonstrates superior performance in semi-supervised segmentation compared to five state-of-the-art semi-supervised methods. Remarkably, our CADNet achieves a mIoU of 46.89% and a F1-score of 63.84%, while all the other models produce collapsed results. Our ablation study demonstrated the effectiveness of the proposed components.

This breakthrough suggests that the mechanism of modeling change is more effective than those appearance-modeling based semantic segmentation methods. Moreover, the change-aware mechanism endows our model with considerable potential for real-world applications, especially in scenarios where appearances are highly variable. Thanks to the efficient change-modeling architecture, both the computational cost and label requirement of CADNet are relative low. This enables the feasibility of our method for developing a streamlined model for basic industrial inspections using only a few samples.

The limitations and future research of our work are as follows: (1) One limitation of our model is its sensitivity to large geometric misalignment, which may impact its performance in real-world scenarios where defects might be detected under non-ideal conditions, such as varying perspectives or misaligned sensor data. This requires further research to address the alignment of heterogeneous data effectively. (2) While we have utilized certain data augmentation strategies, our model’s robustness could be further enhanced by exploring more advanced techniques. For instance, the use of diffusion models to synthetically expand the defect dataset could improve generalization to unseen defect types. This avenue of research is still unexplored in our current work and represents a key area for further development. (3) Although our approach shows promising accuracy results with relatively low computational cost, it can be computationally intensive in a real industry environment, particularly for large-scale datasets. Future work can focus on optimizing the model’s efficiency through techniques such as pruning or hardware acceleration.

## Data Availability

All the datasets utilized in this study are available at https://github.com/HATFormer/CADNet.
